# Effects of glucose-dependent insulinotropic polypeptide on gastric emptying, glycaemia and insulinaemia during critical illness: a prospective, double blind, randomised, crossover study

**DOI:** 10.1186/s13054-014-0718-3

**Published:** 2015-01-23

**Authors:** Palash Kar, Caroline E Cousins, Christopher E Annink, Karen L Jones, Marianne J Chapman, Juris J Meier, Michael A Nauck, Michael Horowitz, Adam M Deane

**Affiliations:** Intensive Care Unit, Level 4, Emergency Services Building, Royal Adelaide Hospital, North Terrace, Adelaide, South Australia 5000 Australia; Discipline of Medicine, The University of Adelaide, Royal Adelaide Hospital, Level 6 Eleanor Harrald Building, North Terrace, Adelaide, South Australia 5000 Australia; Centre of Research Excellence in Translating Nutritional Science to Good Health, The University of Adelaide, Level 6, Eleanor Harrald Building, North Terrace, Adelaide, South Australia 5000 Australia; Discipline of Acute Care Medicine, The University of Adelaide, North Terrace, Adelaide, South Australia 5000 Australia; Diabetes Division, Department of Medicine I, St. Josef-Hospital, Ruhr-University Bochum, Gudrunstraße 56, Bochum, 44791 Germany; Diabetes Centre, Bad Lauterberg, Kirchberg 21, Bad Lauterberg, Harz 37431 Germany

## Abstract

**Introduction:**

Insulin is used to treat hyperglycaemia in critically ill patients but can cause hypoglycaemia, which is associated with poorer outcomes. In health glucose-dependent insulinotropic polypeptide (GIP) is a potent glucose-lowering peptide that does not cause hypoglycaemia. The objectives of this study were to determine the effects of exogenous GIP infusion on blood glucose concentrations, glucose absorption, insulinaemia and gastric emptying in critically ill patients without known diabetes.

**Methods:**

A total of 20 ventilated patients (Median age 61 (range: 22 to 79) years, APACHE II 21.5 (17 to 26), BMI 28 (21 to 40) kg/m^2^) without known diabetes were studied on two consecutive days in a randomised, double blind, placebo controlled, cross-over fashion. Intravenous GIP (4 pmol/kg/min) or placebo (0.9% saline) was infused between T = −60 to 300 minutes. At T0, 100 ml of liquid nutrient (2 kcal/ml) containing 3-O-Methylglucose (3-OMG), 100 mcg of Octanoic acid and 20 MBq Tc-99 m Calcium Phytate, was administered via a nasogastric tube. Blood glucose and serum 3-OMG (an index of glucose absorption) concentrations were measured. Gastric emptying, insulin and glucagon levels and plasma GIP concentrations were also measured.

**Results:**

While administration of GIP increased plasma GIP concentrations three- to four-fold (T = −60 23.9 (16.5 to 36.7) versus T = 0 84.2 (65.3 to 111.1); *P* <0.001) and plasma glucagon (iAUC_300_ 4217 (1891 to 7715) versus 1232 (293 to 4545) pg/ml.300 minutes; *P* = 0.04), there were no effects on postprandial blood glucose (AUC_300_ 2843 (2568 to 3338) versus 2819 (2550 to 3497) mmol/L.300 minutes; *P* = 0.86), gastric emptying (AUC_300_ 15611 (10993 to 18062) versus 15660 (9694 to 22618) %.300 minutes; *P* = 0.61), glucose absorption (AUC_300_ 50.6 (22.3 to 74.2) versus 64.3 (9.9 to 96.3) mmol/L.300 minutes; *P* = 0.62) or plasma insulin (AUC_300_ 3945 (2280 to 6731) versus 3479 (2316 to 6081) mU/L.300 minutes; *P* = 0.76).

**Conclusions:**

In contrast to its profound insulinotropic effect in health, the administration of GIP at pharmacological doses does not appear to affect glycaemia, gastric emptying, glucose absorption or insulinaemia in the critically ill patient.

**Trial registration:**

Australian New Zealand Clinical Trials Registry ACTRN12612000488808. Registered 3 May 2012.

## Introduction

Hyperglycaemia frequently occurs in the critically ill patient, is exacerbated by feeding, and is associated with adverse outcomes [1,2]. Outcomes appear particularly poor in patients without pre-existing diabetes, which accounts for the majority of critically ill patients with hyperglycaemia [1,3-6]. When blood glucose concentrations are elevated, current guidelines recommend administering exogenous insulin, which is associated with substantial risks of hypoglycaemia and perturbations in blood glucose [4,7,8]. Both hypoglycaemia and glycaemic variability may be more harmful than hyperglycaemia [9-11]. Accordingly, for hyperglycaemic critically ill patients who are not known to have diabetes there is a compelling rationale to maintain blood glucose within a narrow range that does not cause hypoglycaemia and limits blood glucose variability [4,12].

The incretin effect refers to the greater insulinotropic response to an oral/enteral glucose load when compared with an intravenous glucose load. The incretin effect is accounted for by incretin hormones, glucagon-like peptide-1 (GLP-1) and glucose-dependent insulinotropic polypeptide (GIP), which are secreted from the small intestine in response to nutrient exposure [4]. GLP-1 stimulates insulin and suppresses glucagon secretion [13]. GIP is also insulinotropic but, in contrast, may stimulate glucagon secretion, particularly at a lower blood glucose level [14]. Importantly, the effects of GLP-1 and GIP are glucose dependent, so that exogenous administration of GLP-1 and/or GIP, even at pharmacological doses, does not cause hypoglycaemia [14]. For this reason there is considerable interest in the potential use of GLP-1 and GIP in the management of hyperglycaemia in the critically ill patient [4,15].

Our group has reported that exogenous GLP-1 retains its potent glucose-lowering effect in the critically ill patient during enteral feeding because it stimulates insulin secretion and slows gastric emptying [16-18]. Slower gastric emptying may be undesirable, however, particularly in relation to the potential to exacerbate gastroesophageal reflux [17] and compromise enteral feeding [19,20].

In health, physiological doses of GIP (~1 pmol/kg/minute) are well tolerated and pharmacological doses (≥1.5 pmol/kg/minute) may accelerate gastric emptying [21], with even greater doses (~4 pmol/kg/minute) having potent insulinotropic effects [22-24]. Additionally, GIP may promote weight gain via increased glucose absorption and/or a trophic effect on adipose tissue [25].

The effects of GIP on insulin and glucagon are affected acutely by perturbations in glycaemia. For example, at normal (6 to 10 mmol/l) and low (≈2.5 mmol/l) blood glucose concentrations, exogenous GIP stimulates glucagon secretion and has negligible effects on insulin secretion; whereas at elevated (≥12.0 mmol/l) blood glucose concentrations, GIP appears to have no effect on glucagon secretion and is profoundly insulinotropic [22,26]. Given that GIP has a bi-directional glucose-dependent effect on glucagon secretion and has been reported to have a stabilising effect on glycaemia in patients with type 2 diabetes [27], exogenous GIP could potentially reduce glycaemic variability in this cohort.

Our group has reported that, in the critically ill patient, GIP at a dose considered slightly above postprandial physiological concentrations (2 pmol/kg/minute) when administered with another potent insulinotropic hormone, GLP-1, does not have an additive glucose-lowering effect [20]. However, the effects of GIP when administered as a sole agent at doses that are pharmacological in this group are unknown. Given that GIP may have a more favourable effect profile on gastric emptying and glucose absorption, it is important to determine the effects of GIP in the critically ill patient.

We hypothesised that exogenous GIP will lower fasting and nutrient-stimulated glycaemia by stimulating insulin secretion, while modestly accelerating gastric emptying, and increasing the rate of glucose absorption. The objectives of this study were to determine the acute effects of exogenous GIP (4 pmol/kg/minute) on glycaemia, gastric emptying, glucose absorption, and insulin secretion during enteral nutrition in patients with acute critical illness-associated hyperglycaemia.

## Methods

### Subjects

Critically ill patients without known diabetes, with blood glucose concentration >7.1 mmol/l when fasting and/or >10 mmol/l during enteral feeding, and who were expected to remain mechanically ventilated via a tracheal tube for at least 48 hours were studied between April and December 2012. All patients had an arterial catheter *in situ*, which is routine care for ventilated patients admitted to the Royal Adelaide Hospital Intensive Care Unit, and this was used for blood sampling. Patients were excluded due to pregnancy, anaemia (haemoglobin <80 g/l), age (<18 years), contraindication to enteral feeding, previous surgery on the small intestine or any gastrointestinal surgery during their then current hospital admission.

### Protocol

**Figure 1:**
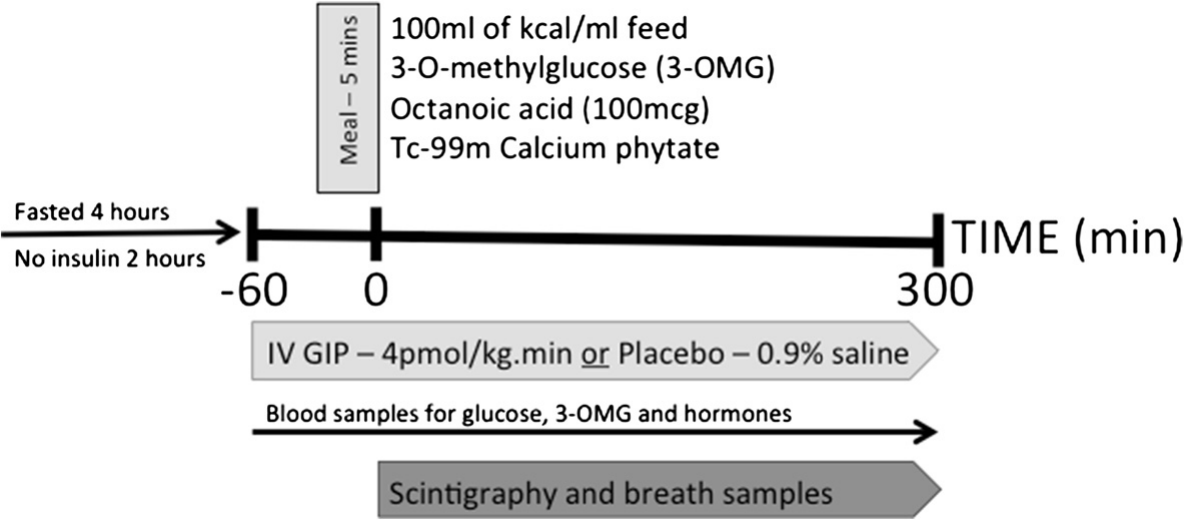
**Protocol of the study.** GIP, glucose-dependent insulinotropic polypeptide; IV, intravenous; mcg, micrograms.

This was a prospective, randomised, double-blind, crossover study. Patients were studied on two consecutive days, on which they received intravenous GIP (4 pmol/kg/minute) or placebo (0.9% saline) at the commencement of the study period (T–60) (Figure 1). Patients were fasted for 4 hours and exogenous insulin (Actrapid) was ceased 2 hours prior to each study. Patient weight was measured using bed scales (MPWS; A&D Medical, Sydney, NSW, Australia). Synthetic GIP (Bachem, Weil am Rhein, Germany) was reconstituted by the Royal Adelaide Hospital Department of Pharmacy in 0.9% saline. The Department of Pharmacy was also responsible for computer-generated randomisation. While study drugs appeared identical, treatment blinding was ensured by the use of plastic coverings over all solutions. Study drugs were delivered through low-absorbance tubing (Verasafe; Carefusion, San Diego, CA, USA) to prevent protein binding [16-18]. The randomisation schedule was kept in a locked facility within the Department of Pharmacy and the investigators had no access to the schedule during the study period. All solutions were given via a central venous catheter at 1 ml/minute using an infusion pump (Alaris; Cardinal Health, Sydney, NSW, Australia). Sixty minutes after the study drug was commenced (that is, at T0), a liquid nutrient meal was administered via nasogastric tube over 5 minutes. The meal contained 100 ml TwoCal® (2 kcal/ml; Abbot Nutrition, Botany, NSW, Australia), a mixed nutrient liquid containing carbohydrate (43%), fat (40%), and protein (17%), as well as 3 g 3-*O*-methyglucose (3-OMG; Sigma-Aldrich, Sydney, NSW, Australia) dissolved in 5 ml water, 100 μg octanoic acid (Sigma-Aldrich), and 20 MBq technetium-99 m calcium phytate (Radpharm Scientific, Belconnen, ACT, Australia). Patients were studied for 360 minutes (from T–60 to T300) in total during each study period.

This study was approved by the Research Ethics Committee of the Royal Adelaide Hospital and the protocol was registered with the Australian New Zealand Clinical Trials Registry (ACTRN number 12612000488808). Patients were unconscious when enrolled and consent was therefore obtained from and signed by their next of kin.

### Data collection

Arterial blood samples (5 ml) were collected immediately prior to administration of the study drug (T–60) and the intragastric meal (T0), and at 15-minute intervals from T0 to T60, and then at 30-minute intervals until T300, for measurements of serum 3-OMG and blood glucose concentrations. Samples for measurement of serum insulin were collected at T–60, 0, 15, 30, 45, 60, 90, 120, 150, 180, 210, 240 and 300 minutes, for serum glucagon at T–60, 0, 30, 60, 120, 180, 240 and 300 minutes, and for plasma GIP at T–60, 0, 60, 120 and 300 minutes. Blood was stored in ice at all times. Serum was separated by centrifugation within 30 minutes of completion of the study (3,200 rpm for 15 minutes at 4°C) and then stored at –70°C until assayed. Expiratory breath samples were collected as described previously [17]. Left anterior oblique (45°) images were acquired using a mobile gamma camera (Digirad, Poway, CA, USA) in 3-minute dynamic frames from T0 to T300 with patients positioned supine [28].

### Blood glucose, glucose absorption and insulin, glucagon and glucose-dependent insulinotropic polypeptide

Blood glucose concentrations were measured and recorded immediately, by the investigators, using a blood gas analyser (ABL800 FLEX; Radiometer, Copenhagen, Denmark) [20]. The monosaccharide 3-OMG is absorbed from the small intestine via the same transporters as glucose, but is not metabolised [28,29], and measurement of serum 3-OMG concentrations provides an accurate measure of glucose absorption in healthy individuals and the critically ill [28,29]. Serum 3-OMG concentrations were measured using liquid chromatography/mass spectroscopy, with an assay sensitivity of 0.0103 mmol/l [17]. When the baseline (T–60) serum concentrations of 3-OMG on day 2 were greater than the assay sensitivity (that is, fasting serum 3-OMG concentration >0.0103 mmol/l), the concentration at T–60 was referenced as zero for subsequent analysis [28].

Serum insulin was measured by enzyme-linked immunosorbent assay (10-1113; Mercodia, Uppsala, Sweden), which had an interassay coefficient of variation of 5.4% and an intraassay coefficient of variation of 2.7 [20]. Serum glucagon was measured via radioimmunoassay (GL-32 K; Millipore, Billerica, MA, USA). The minimum detectable limit was 20 pg/ml, with an interassay coefficient of variation of 6.1% and an intraassay coefficient of variation of 4.1% [18]. Plasma total GIP was measured by radioimmunoassay (Perkin Elmer, Boston, MA, USA), with an interassay coefficient of variation of 8.3% and an intraassay coefficient of variation of 6.3% [20].

### Glycated haemoglobin

Glycated haemoglobin was determined using high-performance liquid chromatography [20]. Unrecognised diabetes was defined as glycated haemoglobin >6.5% (48 mmol/mol) in patients with no history of diabetes [6].

### Gastric emptying

Gastric emptying was measured using two different techniques: scintigraphy – although the gold standard, this technique requires the availability of both a mobile gamma camera and a trained nuclear medicine technologist, and these could not be guaranteed to be available on every study day; and radioisotope (^13^C-octanoic breath test), which was available for every study day.

Gastric scintigraphy requires mixing of a radioisotope (20 MBq technetium-99m calcium phytate) with a meal that is administered via nasogastric tube. A gamma camera then records images of the labelled meal, which indicates the percentage of the meal remaining within the stomach at any time point. The greater the percentage retained within the stomach, the slower gastric emptying. Scintigraphic data were analysed by a nuclear medicine technologist (KLJ) blinded to the study conditions. Radioisotopic data were corrected for subject movement and radionuclide decay. A region of interest was drawn around the total stomach, gastric emptying curves generated over time, and intragastric retention derived at 15-minute intervals from T0 to 300 minutes.

The ^13^C-octanoic breath test was performed as described previously [17,28]. Data were expressed as the gastric emptying coefficient, a global measure of gastric emptying; a higher number is indicative of more rapid emptying [17].

### Statistical analysis

The sample size was based on calculations that 20 patients would provide 80% power, at a two-sided α-level of 0.05, to detect a minimum difference in postprandial glycaemia (glucose levels in the blood) of 290 mmol/l. 300 minutes between groups, which was predefined as clinically significant, and was based on the within-patient standard deviation of glycaemia as mmol/l.300 minutes [17].

While differences between GIP and placebo were distributed normally, most of the raw data were skewed. Accordingly, all data are presented as median (range or 25th to 75th percentile), unless specified otherwise. Significance was determined using nonparametric Wilcoxon signed-rank tests. Serum 3-OMG (glucose absorption), plasma insulin and blood glucose concentrations are presented as areas under the concentration curve (AUCs), and were calculated using the trapezoidal rule. Relative glucagon response was measured by the incremental AUC using the trapezoidal rule. The absolute glucagon change from baseline was used to remove intrasubject variation in baseline levels. The maximal effects of gastric emptying were anticipated to occur in the first 60 minutes after the meal, so this period was also chosen *a priori* for analyses. All reported *P* values are two sided, with the 0.05 level selected to determine significance. When significant, multiple comparisons were adjusted for using the Bonferroni–Holm procedure. Data were evaluated for potential carry over and/or period effects by including the order variable in repeated-measures analysis of variance; however, there were no order-by-treatment interactions. Between-subject Pearson correlations were calculated on each study visit separately between the initial rate of gastric emptying (% gastric retention at T = 60 minutes as determined using scintigraphy) and each of glycaemia, insulin and 3-OMG absorption (the delta value from 0 to 60 minutes for each). Scatter plots were examined to assess the linearity of the relationship and Pearson’s correlation was considered appropriate in each case. Steiger’s *Z*_2_* test for difference between two dependent correlations was used to compare the correlations between the same outcomes between the two visits. Statistical analyses were performed using SPSS (Version 18.0) was supplied by: "The University of Adelaide, Adelaide, SA, Australia". An independent professional biostatistician had access to all data and verified these analyses.

## Results

Twenty-four patients were enrolled and no adverse effects (vomiting, hypoglycaemia, seizure or rash) were observed with the study drug. Blood results were also reviewed with no unexpected changes to haemoglobin, platelets, liver function tests and electrolytes. Four patients failed to complete both study days due to tracheal extubation (two patients), withdrawn consent (one patient) and migration of the feeding tube into the small intestine (one patient). Data from these patients were not included in the analyses. Demographic details for patients completing the study are summarised in Table 1. One patient was diagnosed with unrecognised diabetes with a glycated haemoglobin of 9.1% (76 mmol/mol). Peak fasting and peak postprandial glucose concentrations along with administered medications, sedation score and temperature were also recorded (Table 1).

**Table 1:** **Patient characteristics**

Age (years)	62 (22 to 79)
Sex	Male: 12, female: 8
Body mass index (kg/m^2^)	28 (21 to 40)
APACHE II (score)	21.5 (17 to 26)
Length of ICU admission prior to study day 1 (days)	3.0 (1 to 16)
Glycated haemoglobin (HbA1c)	
%	5.9 (5.3 to 9.1)
mmol/mol	40.5 (34 to 76)
Calories delivered in previous 24 hours (kcal)	885 (0 to 1,680)
Feed tolerant (patients)	14
Blood glucose concentration (mmol/l)	
Peak fasting	9.5 (6.6 to 14.2)
Peak post prandial	10.7 (7.9 to 17.9)
Medications^a^	
Catecholamines	10
Noradrenalin	10
Adrenalin	1
Opiates	11
Fentanyl	10
Oxycodone	1
Sedatives	16
Propofol	14
Midazolam	3
Ketamine	1
Dexmedetomidine	1
Insulin	8
Peak dose (units/hour)	5.5 (2.5 to 10.5)
Dose in previous 24 hours (units)	42.4 (15.0 to 117.0)
Corticosteroid	7
Hydrocortisone dose (or equivalent) on study day (mg/day)	200 (50 to 1,000)
RASS sedation score	−4 (−2 to –5)
Body temperature (°C)	37.0 (31.7 to 38.5)
Diagnosis category	
Neurology	6
Trauma	4
Respiratory	3
Sepsis	2
Cardiovascular	2
Other	3

Data presented as median (range); *n* = 20. APACHE, Acute Physiology and Chronic Health Evaluation; RASS, Richmond Agitation Sedation Scale. ^a^Patients were on multiple medications during the course of the study.

### Blood glucose, glucose absorption and hormones

**Figure 2:**
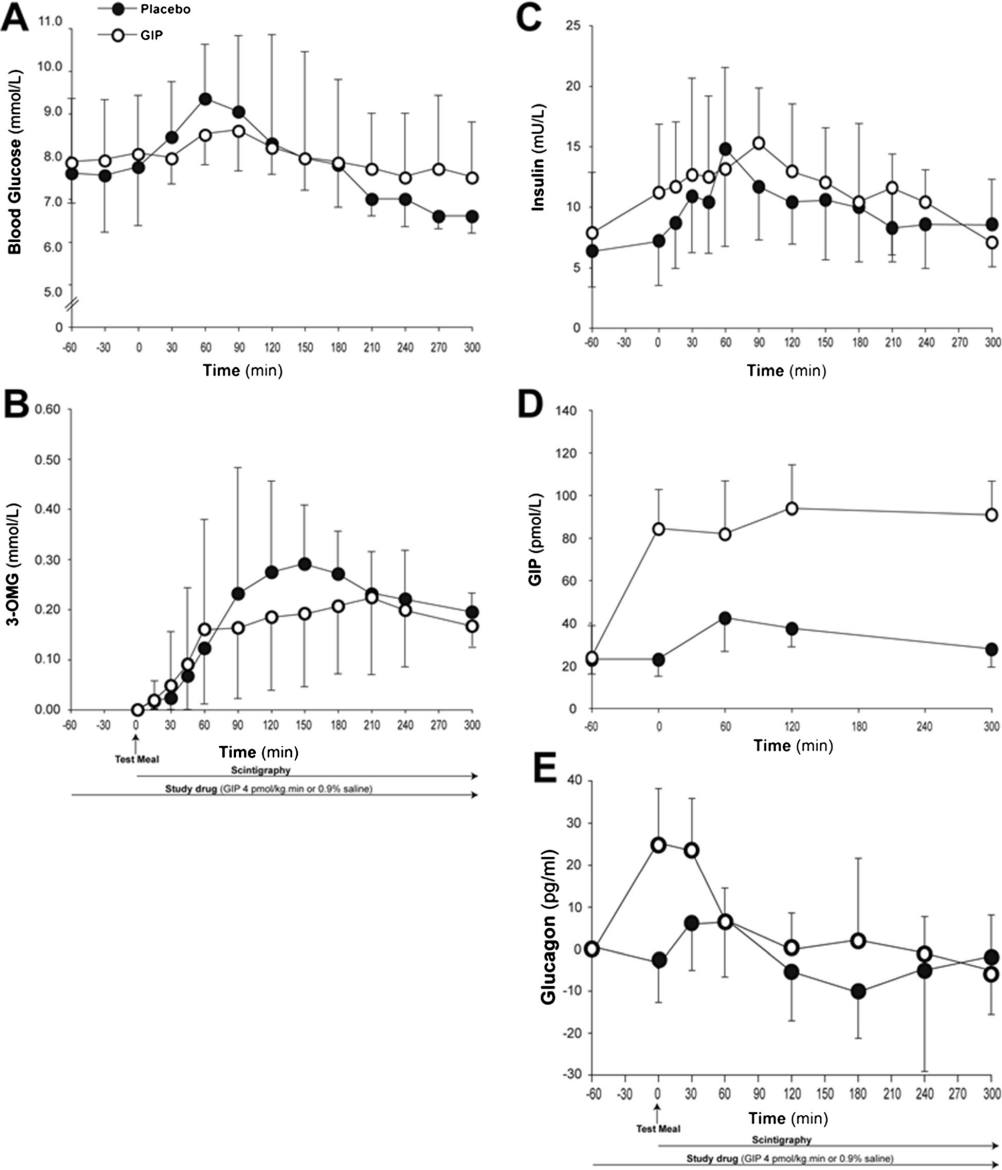
**Effects of glucose-dependent insulinotropic polypeptide.** Effects of glucose-dependent insulinotropic polypeptide (GIP; 4 pmol/kg/minute) on: **(A)** glycaemia (AUC_–60 to 300_: GIP, 2,843 (2,468 to 3,639) vs. control, 2,819 (2,578 to 3,788) mmol/l.300 minutes; *P* = 0.86); **(B)** glucose absorption (serum 3-O-methylglucose (3-OMG)) (AUC_0 to 300_: 50.6 (22.3 to 74.2) vs. 64.3 (9.9 to 96.3) mmol/l.300 minutes; *P* = 0.62); **(C)** insulin concentrations (AUC_–60 to 300_: 3,945 (2,280 to 6,731) vs. 3,479 (2,499 to 5,658) mU/l.300 minutes; *P* = 0.76); **(D)** GIP concentrations (*P <0.001; Bonferroni–Holm correction for all time points); **(E)** glucagon concentrations (incremental AUC_–60 to 300_: 4,217 (1,891 to 7,715) vs. 1,232 (293 to 4,545) pg/ml.300 minutes; *P* = 0.04). Data are median (25th to 75th percentile), analysed using Wilcoxon signed-rank test; n = 20. AUC, area under the concentration curve.

Baseline blood glucose concentrations were similar on both days (at T − 60: GIP 7.5 (6.5 to 9.5) vs. control 7.6 (7.0 to 9.4) mmol/l; *P* = 0.68). GIP had no effect on blood glucose before the meal (at T0: 8.1 (9.6 to 9.0) vs. 7.8 (6.8 to 9.0) mmol/l; *P* = 0.53). There was a rise in blood glucose concentration after the meal (Figure 2A), peaking between 60 and 90 minutes, but GIP had no effect on either peak glucose concentrations (9.4 (8.3 to 11.9) vs. 9.8 (8.4 to 11.8) mmol/l; *P* = 0.73) or the overall glycaemic response (AUC_300_: 2,843 (2,568 to 3,338) vs. 2,819 (2,550 to 3,497) mmol/l.300 minutes; *P* = 0.86). Data were similar when the patient with unrecognised diabetes was excluded (AUC_300_: 2,991 (2,469 to 3,639) vs. 2,781 (2,578 to 3,738) mmol/l.300 minutes *P* = 0.74). Glucose absorption was unaffected by GIP administration (AUC_300_: 50.6 (22.3 to 74.2) vs. 64.3 (9.9 to 96.3) mmol/l.300 minutes; *P* = 0.62) (Figure 2B).

Insulin concentrations were similar at baseline on both study days (at T − 60: 7.9 (4.8 to 12.0) vs. 6.4 (2.9 to 13.5) mU/l; *P* = 0.75). There was a postprandial rise in insulin concentrations, peaking between 60 and 90 minutes. Overall insulin response was not affected by GIP (AUC_300_: 3,945 (2,280 to 6,731) vs. 3,479 (2,316 to 6,081) mU/l.300 minutes; *P* = 0.76) (Figure 2C). Plasma GIP concentrations were comparable at baseline (at T − 60: 23.9 (16.5 to 36.7) vs. 23.0 (15.6 to 41.9) pmol/l; *P* = 0.96) and the exogenous GIP infusion resulted in a threefold to fourfold increase above physiological concentrations (*P* <0.001, Figure 2D).

Glucagon concentrations were also similar at baseline (at T − 60: 104.5 (85.1 to 236.6) vs. 115.7 (85.8 to 287.6) pg/ml; *P* = 0.37) and prior to the meal (at T0: 128.5 (99.4 to 290.8) vs. 112.5 (82.8 to 292.9) pg/ml; *P* = 0.08). However, the postprandial increment was significantly increased with GIP as compared with control (incremental AUC_300_: 4,217 (1,891 to 7,715) vs. 1,232 (293 to 4,545) pg/ml.300 minutes; *P* = 0.04) (Figure 2E).

### Gastric emptying

Paired scintigraphic data were collected in 18 patients and breath test data were available for all patients. Using scintigraphy, 100% of the meal remained in the stomach at T = 300 minutes in one patient on both study days and in two other patients during either GIP or placebo, indicative of markedly delayed gastric emptying.

**Figure 3:**
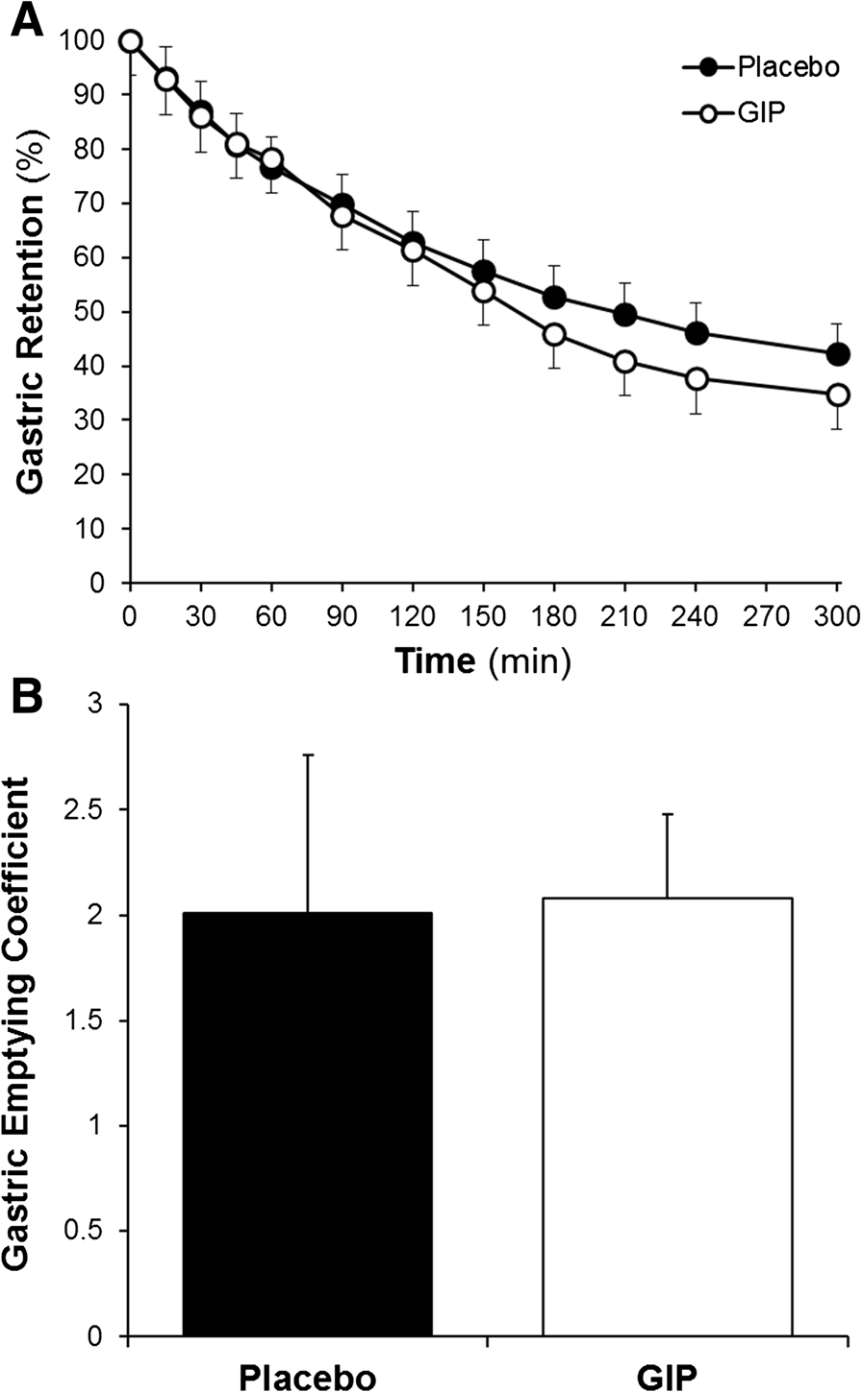
**Gastric emptying.** Effect of glucose-dependent insulinotropic polypeptide (GIP) on gastric emptying as measured using: **(A)** retention of gastric contents over time (scintigraphic technique) (AUC_0 to 300_: GIP, 15,611 (10,993 to 18,062) vs. placebo, 15,660 (9,694 to 22,618)%.300 minutes; *P* = 0.61; *n* = 18); and **(B)** gastric emptying coefficient (labelled breath test) (1.98 (1.60 to 2.50) vs. 2.01 (1.14 to 2.81); *P* = 0.99; *n* = 20). Data are median (25th to 75th percentile); analysed using Wilcoxon’s signed-rank test. AUC, area under the concentration curve.

GIP had no effect on intragastric retention 60 minutes after the meal (at T60: 80 (66 to 89) vs. 84 (60 to 96)%; *P* = 0.88) and at the study end (at T300: 26 (10 to 63) vs. 37 (7 to 92)%; *P* = 0.33), or on the overall gastric emptying rate as determined using scintigraphy and breath test techniques (Figure 3A,B).

### Relationships

**Figure 4:**
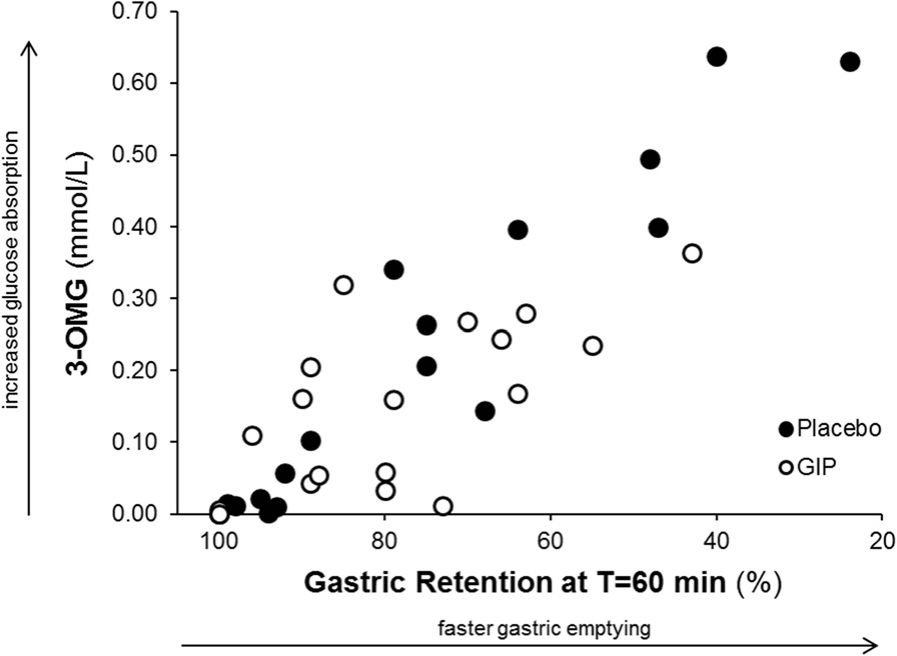
**Relationship between glucose absorption and gastric emptying.** Relationship between 3-O-methylglucose (3-OMG) concentrations (glucose absorption) and gastric emptying (retention at T = 60; scintigraphy) during glucose-dependent insulinotropic polypeptide (GIP; *r* = 0.66; *P* <0.01) and placebo (*r* = 0.95; *P* <0.01). Data are analysed between subjects using Pearson correlations; *n* = 18.

The change in blood glucose was related to gastric emptying; the more rapid the emptying, the greater the glycaemic excursion during placebo (*r* = 0.85; *P* <0.01) and GIP (*r* = 0.48; *P* = 0.04), with the correlation significantly stronger during placebo (*z* = 2.1; *P* = 0.04). There was a close relationship between 3-OMG concentrations (glucose absorption) and gastric emptying during both placebo and GIP (Figure 4). However, the relationship was significantly stronger during placebo (*z* = 3.1, *P* <0.01). Relatively more rapid gastric emptying was also associated with increased insulin secretion during placebo (*r* = 0.48; *P* = 0.04) and GIP (*r* = 0.47; *P* <0.05), with no difference between placebo and GIP (*z* = 0.02, *P* = 0.98).

## Discussion

This study indicates in patients with critical illness-associated hyperglycaemia that acute intravenous administration of GIP at pharmacological doses has no insulinotropic activity, does not reduce elevated blood glucose concentrations but does cause a significant postprandial rise in glucagon.

The mechanism(s) underlying the absence of a glucose-lowering effect of GIP are uncertain. Based on the known effects of GIP in ambulant populations, the preceding acute glycaemic disturbance associated with critical illness is probably important. Chronic hyperglycaemia has been shown to profoundly diminish the insulinotropic effect of GIP; that is, the insulinotropic effect is almost abolished in patients with longstanding hyperglycaemia [24,30,31]. In patients with type 2 diabetes, 4 weeks of intensive insulin therapy aiming for near-normal glycaemia partially re-established the insulinotropic properties of GIP [32]. While the duration of normoglycaemia or hyperglycaemia required to modify the response to GIP in humans remains to be determined, in cell cultures as little as 24 hours exposure to glucose concentrations >11 mmol/l leads to a substantial downregulation of GIP receptors on beta cells [33]. However, with respect to this study, in the absence of data from patients without hyperglycaemia, this hypothesis is difficult to prove.

The critically ill patients in this study were studied relatively early in their admission and the objective prior to the intervention was to restrict glucose concentrations to <10 mmol/l while in the ICU. These features are consistent with the concept that the magnitude and duration of hyperglycaemia required to attenuate the insulinotropic effect of GIP in humans is relatively modest. While hyperglycaemia may possibly be an important modulator, the possibility that the response to GIP is caused by critical illness *per se* cannot be excluded. Increased secretion of cytokines and other counter-regulatory hormones are prominent features of critical illness-associated hyperglycaemia [1,4] and these cytokines possibly downregulate responsiveness to GIP in the critically ill independent of hyperglycaemia.

Other reasons for the lack of glucose-lowering effect may be due to the effect of GIP on glucagon. Exogenous GIP is known to be glucagonotropic at normal and low blood glucose concentrations, and therefore the rise in levels of glucagon within this study may have contributed to the absence of blood glucose lowering.

It has been reported in healthy subjects that exogenous GIP attenuates postprandial glycaemia while mildly accelerating gastric emptying [21], but in this study gastric emptying was unaffected by GIP during critical illness. A possible explanation for this difference is that the acceleration of gastric emptying observed in the former study may have resulted from the insulinotropic effects of GIP, which, by lowering blood glucose concentrations, had a mild gastrokinetic effect, given that systemic glycaemia is a major determinant of the emptying rate [34]. However, blood glucose concentrations were unaffected in the study population and therefore the effect of GIP on the gastric emptying rate was expected to be somewhat marginal.

The effect of GIP on nutrient absorption in the critically ill was of particular interest. Glucose absorption is markedly diminished in the critically ill patient and downregulation of the sodium–glucose co-transporter 1 appears to be pivotal [29,35]. In isolated mice jejunum, GIP increases glucose transport across the lumen, via upregulation of sodium–glucose co-transporter 1 [25]. In this study, glucose absorption did not appear to be affected by GIP. However, an effect of GIP on nutrient absorption cannot be completely dismissed because nutrient was delivered into the stomach and small intestinal nutrient absorption can only be accurately measured when nutrient is delivered distal to the pylorus [35]. The relationship between glucose absorption and gastric emptying being weaker during GIP suggests that factors distal to the pylorus may be relevant. Furthermore, the study period was relatively short and may have been insufficient to detect any effect of sodium–glucose co-transporter 1 expression and subsequent functional (absorptive) outcomes.

A particular strength of this study is that the cohort had features consistent with acutely impaired glucose tolerance, although one patient was subsequently shown to have unrecognised type 2 diabetes. In addition, median blood glucose concentrations were ~8 mmol/l, which should have been sufficient to stimulate an insulinotropic effect of GIP [13]. For these reasons it is likely that the lack of effect observed represents a true observation.

There are, however, limitations to this study. Only a single dose of GIP was tested, and it cannot be assumed that glycaemia will remain unaffected at greater doses. However, GIP administered at 4 pmol/kg/minute has substantial biological effects in both healthy individuals and patients with diabetes, consistent with the concept that this amount reflects a potent pharmacological dose [21,22,24,31,36,37] – and even at one-half the dose administered in this study (2 pmol/kg/minute) GIP accelerates gastric emptying [21], suggesting that the dose chosen was sufficient to have a pharmacological effect. Additionally, insulin levels were measured as opposed to C-peptide, which may be a better marker of endogenous insulin production, as C-peptide analysis was cost prohibitive.

In the current study a profound threefold to fourfold increase in plasma GIP concentrations was evident. This increase in plasma concentration is similar to previous studies where a significant effect has been shown with GIP administration in both healthy individuals [21] and in patients with diabetes [37]. While some studies using a similar dose have reported greater increases in plasma GIP concentrations [24,31], this may well be explained by the different assay techniques. Nonetheless, there is the possibility that achieving greater GIP concentrations may affect glycaemia differently.

The number of patients studied was relatively few, such that it may be underpowered to show a difference in insulin and 3-OMG concentrations. There was also substantial heterogeneity between patients with regards to their diagnosis and the duration of ICU admission. However, the capacity of GLP-1 to affect glycaemia has been observed using smaller cohorts [16,38]. The exposure to exogenous GIP was relatively short (6 hours) and it remains possible, albeit intuitively unlikely, that more prolonged exposure to GIP would reveal an insulinotropic effect.

The patients within this study had only moderate hyperglycaemia. There is a possibility that clamping blood glucose at a higher concentration (for example, 12 mmol/l) may have lead to administration of GIP at 4 pmol/kg/minute, causing a greater insulinotropic effect. Finally, synthetic GIP currently remains an expensive product, which limits its use for proof-of-principle studies.

Along with these limitations, other factors may influence blood glucose levels such as catecholamine infusions, corticosteroid use, depth of sedation and body temperature. These variables are common within the critical care population, and may be confounders with respect to this study.

Several different groups have evaluated the effects of synthetic GLP-1, or its agonists, on glycaemia in the critically ill with – and without – antecedent type 2 diabetes, and there is consistent evidence that GLP-1 has a prominent glucose-lowering effect [16-18,39]. When evaluating this study with our previous observation – that GIP (2 pmol/kg/minute) had no additive insulinotropic effect in the critically ill patient when administered in combination with GLP-1 – it appears that future studies should focus on the use of GLP-1 or its agonists rather than GIP. These observations should not, however, be extrapolated to the potential use of dipeptidyl peptidase-4 inhibitors (which inhibit the enzyme that inactivates GIP and GLP-1) to treat hyperglycaemia in the critically ill patient, because the efficacy of dipeptidyl peptidase-4 inhibitors may result in part from increases in intestinal and portal blood GLP-1 and GIP concentrations [40].

## Conclusions

In critically ill patients, an acute infusion of GIP at 4 pmol/kg/minute had no effect on glycaemia, gastric emptying, glucose absorption, insulin or glucagon secretion. Because the magnitude and duration of hyperglycaemia required to attenuate the insulinotropic effect of GIP appears to be relatively modest, future evaluation of the use of incretin-based approaches in the critically ill patient should focus on GLP-1 and its agonists.

## Key messages

- In the healthy individual, GIP is a potent insulinotropic hormone leading to glucose lowering.
- In the critically ill patient, the effects of GIP are not apparent.

## Abbreviations

AUC: area under the concentration curve
GIP: glucose-dependent insulinotropic polypeptide
GLP-1: glucagon-like peptide 1
3-OMG: 3-*O*-methylglucose
